# Relevance of PSGL-1 Expression in B Cell Development and Activation

**DOI:** 10.3389/fimmu.2020.588212

**Published:** 2020-11-12

**Authors:** Rafael González-Tajuelo, Elena González-Sánchez, Javier Silván, Antonio Muñoz-Callejas, Esther Vicente-Rabaneda, Javier García-Pérez, Santos Castañeda, Ana Urzainqui

**Affiliations:** ^1^ Immunology Department, Fundación de Investigación Biomédica (FIB), Instituto de Investigación Sanitaria-Princesa (IIS-Princesa), Hospital de la Princesa, Madrid, Spain; ^2^ Rheumatology Department, Fundación de Investigación Biomédica (FIB), Instituto de Investigación Sanitaria-Princesa (IIS-Princesa), Hospital de la Princesa, Madrid, Spain; ^3^ Pulmunology Department, Fundación de Investigación Biomédica (FIB), Instituto de Investigación Sanitaria-Princesa (IIS-Princesa), Hospital de la Princesa, Madrid, Spain; ^4^ Cátedra UAM-Roche, EPID-Future, Universidad Autónoma de Madrid (UAM), Madrid, Spain

**Keywords:** PSGL-1 (CD162), B cells, development, activation, pulmonary arterial hypertension

## Abstract

PSGL-1 is expressed in all plasma cells, but only in a small percentage of circulating B cells. Patients with systemic sclerosis (SSc) show reduced expression of PSGL-1 in B cells and increased prevalence of pulmonary arterial hypertension. PSGL-1 deficiency leads to a SSc-like syndrome and SSc-associated pulmonary hypertension in female mice. In this work, the expression of PSGL-1 was assessed during murine B cell development in the bone marrow and in several peripheral and spleen B cell subsets. The impact of PSGL-1 absence on B cell biology was also evaluated. Interestingly, the percentage of PSGL-1 expressing cells and PSGL-1 expression levels decreased in the transition from common lymphoid progenitors to immature B cells. *PSGL-1^−/−^* mice showed reduced frequencies of peripheral B cells and reduced B cell lineage-committed precursors in the bone marrow. In the spleen of WT mice, the highest percentages of PSGL-1^+^ populations were shown by Breg (90%), B1a (34.7%), and B1b (19.1%), while only 2.5–8% of B2 cells expressed PSGL-1; however, within B2 cells, the class-switched subsets showed the highest percentages of PSGL-1^+^ cells. Interestingly, *PSGL-1^−/−^* mice had increased IgG^+^ and IgD^+^ subsets and decreased IgA^+^ population. Of note, the percentage of PSGL-1^+^ cells was increased in all the B cell subclasses studied in peritoneal fluid. Furthermore, PSGL-1 engagement during *in vitro* activation with anti-IgM and anti-CD40 antibodies of human peripheral B cells, blocked IL-10 expression by activated human B cells. Remarkably, PSGL-1 expression in circulating plasma cells was reduced in pulmonary arterial hypertension patients. In summary, although the expression of PSGL-1 in mature B cells is low, the lack of PSGL-1 compromises normal B cell development and it may also play a role in the maturation and activation of peripheral naïve B cells.

## Introduction

B cells are major players in vertebrate adaptive immune responses. They can exert diverse functions such as antibody secretion, cytokine and chemokine production, and antigen presentation. In addition, some B cell subsets can suppress effector T cell proliferation and induce regulatory T cell (Treg) proliferation, acting as regulatory B cells (Bregs) (1–4). Given the importance of B cells for the development of an optimal and fine-tuned immune response, several checkpoints and control mechanisms regulate B cell generation and maturation from bone marrow to secondary lymphoid organs. Additional mechanisms exist to prevent the generation and proliferation of self-reactive B cells that may lead to autoantibody production and autoimmunity (5, 6).

B cell differentiation in adult bone marrow starts with the asymmetric division of a hematopoietic stem cell (HSC) generating a multipotent progenitor (MPP) (7), which no longer possesses self-renewal properties and constitutes the last common progenitor of myeloid and lymphoid lineages (8, 9). The lymphoid lineage is defined by the expression of Flt3, which is first detected in the lymphoid-primed multipotent progenitor. The down-regulation of the stem cell markers Sca-1 and c-KIT and the up-regulation of IL-7 receptor (IL-7R) characterize the common lymphoid progenitor (CLP) (10, 11). After the CLP stage, T cell and B cell lineages are definitely separated into different branches, as T cell precursor exit the bone marrow to reach the thymus. To accomplish B cell generation, B cell precursors must undergo sequential productive DNA rearrangement in the immunoglobulin loci. These rearrangements produce both heavy (Igμ) and light immunoglobulin chains (Igκ, Igλ), which later complete their assembly and subsequent expression as B cell receptors (BCRs). Igμ rearrangement begins in pre-proB cells (12), also called fraction (Fr.) A in the nomenclature of Hardy and Hayakawa (13), which are defined by B220 expression but lack canonical B-cell markers including CD19 (14); the rearrangement continues with the Rag-dependent recombination of variable (V) gene segments to rearranged (D)J regions, which occurs in late pro-B cells (Fr.B and Fr.C) (12, 13). Once the immunoglobulin heavy chain is successfully expressed, the surrogate light chain and the signaling subunits can assemble to form the pre-B cell receptor (pre-BCR), which is expressed on the surface of large pre-B cells (Fr. C’) (12, 13). The pre-BCR promotes the expansion of large pre-B cells, which remain dependent on IL-7 signaling (15, 16). To begin Igκ or Igλ gene rearrangement, pre-B cells must reduce the proliferative signals provided by IL-7R, which are dependent on antagonistic signaling by the pre-BCR (12). Once the recombination of the light chain loci has been accomplished, IgM B-cell receptor (BCR) is expressed on the surface of immature B cells (17), which leave the bone marrow to complete their development in the spleen and join the mature B-cell pool (18). Mature B cells are now able to re-circulate between blood and secondary lymphoid organs. Eventually, the contact with their cognate antigen leads mature B cells to the germinal centers (GC) in the spleen and lymph nodes, where they undergo class switch recombination (CSR) and somatic hypermutation thereby differentiating into high affinity soluble antibody-producing plasma cells (19).

PSGL-1 is an adhesion molecule that binds P-selectin, the main receptor for PSGL-1, and that it is responsible for the tethering and rolling of leukocytes on activated endothelium, which under inflammatory conditions express P-selectin (20). The relevance of PSGL-1/P-selectin interactions in different animal models of acute inflammation has been well established (21–26). However, in recent years, a new role in the preservation of immune homeostasis has been described for PSGL-1/P-selectin interaction (27–30). In human monocyte-derived dendritic cells, interaction between P-selectin and PSGL-1 triggers a tolerogenic program characterized by the increase in IL-10, TGF-β; and IDO mRNA (30). Furthermore, PSGL-1 knock-out mice (*PSGL-1^−/−^*) develop a systemic sclerosis (SSc)-like syndrome recapitulating the hallmarks of human disease: vascular damage, autoimmunity, and skin fibrosis (30). Interestingly, *PSGL-1^−/−^* female mice also develop pulmonary hypertension associated with this scleroderma-like syndrome (27). In the case of P-selectin deficient mice (*P-sel^−/−^*), lack of PSGL-1/P-selectin interaction results in a systemic lupus erythematosus (SLE)-like syndrome, characterized by the production of SLE-specific autoantibodies such as anti-dsDNA or anti-RNP, reduced dermal T effector/Treg ratio, and lung and renal involvement (28). Remarkably, in humans, up to 60% of the IL-10-producing B cells express PSGL-1, pointing to a regulatory role for PSGL-1 in B cells (31). Interestingly, PSGL-1 expression is decreased in peripheral blood B cells from SSc patients compared with age-matched healthy controls and these PSGL-1^+^ B cells from SSc patients present decreased IL-10 production. Moreover, PSGL-1 fails to induce Syk phosphorylation and IL-10 production in monocytes from SSc patients after interaction with P-selectin, thus highlighting the loss of PSGL-1 regulatory role in SSc (31).

In this work, we sought to characterize the expression pattern of PSGL-1 during B cell generation and maturation, analyzing cell numbers, relative frequency, and PSGL-1 expression level in different B cell subpopulations, from bone marrow early multipotent progenitors to splenic mature B cells. Taking into account the regulatory role of PSGL-1 in mice and humans, and the higher susceptibility to PAH and lower PSGL-1expression on B cells found in patients with SSc, we also investigated PSGL-1 expression on B cells and plasma cells from PAH patients and healthy controls. Additionally, we evaluated whether PSGL-1 signaling in human B cells could control their *in vitro* activation.

## Materials and Methods

### Mice

C57BL/6 *PSGL-1^−/−^*mice were kindly provided by Dr. M. K. Wild and Dr. D. Vestweber (Max Planck Institute for Molecular Biomedicine, Münster, Germany). Wild-type (WT) C57BL/6 mice were obtained from The Jackson Laboratory and were backcrossed with *PSGL-1^−/−^*mice to homogenize the genotype. Genotyping of WT and KO mice was performed by PCR, using the following primers: Forward (5’ - gag ggt aag gaa cct tct ctg atg - 3’), Reverse WT (5’ - tgg atg ctg gtt gga cgg tc - 3’), and Reverse KO (5’ - gtc acg tcc tgc acg acg c - 3’).

Mice were kept in pathogen-free conditions at the Animal Facility of the School of Medicine, Universidad Autónoma de Madrid. Male and female mice aged 3–5 months were used indistinctly for the experiments, since we did not find substantial differences between sexes in the variables studied in this work. Mice were killed by cervical dislocation, and internal organs were extracted for analysis. All experiments and breeding were performed in accordance with national and institutional guidelines for animal care (EU Directive 2010/63/EU for animal experiments). The experimental procedures were approved by the Director General de Medio Ambiente of Madrid (ref. PROEX 69/14 and PROEX 162/15).

### Obtainment and Processing of Blood and Organs

For flow cytometry experiments, after mouse dissection spleens were kept in cold PBS 1X until use. Then, spleens were minced with the frosted end of glass microscope slides, and the disaggregated cell suspension was washed twice and filtered with a 30-μm cell strainer.

Bone marrow was obtained from the tibia and femur, by perfusion of the diaphysis with PBS 1X BSA 0.5% with a 21G needle. The cell suspension was homogenized by soft pipetting and washed twice. Then, cells were filtered with a 30-μm cell strainer.

Blood was extracted by cardiac puncture after sacrifice, using a heparinized needle and syringe. Peritoneal cells were extracted by perfusion of the peritoneal cavity with 4 ml of PBS 1X. After that, PBS 1X containing the peritoneal cells was recovered and washed twice with PBS 1X BSA 0.5% EDTA 0.5mM.

For histological samples, spleens were fixed in 4% paraformaldehyde for 48 h. After fixation, spleens were dehydrated, embedded in paraffin, and cut into 4-μm sections. Spleen sections were stained with hematoxylin and eosin and analyzed with a brightfield microscope.

### Human B Cell Extraction and Culture

Human blood samples of anonymous healthy donors were obtained from buffy coats provided by the Centro de Transfusiones de la Comunidad de Madrid, following international recommendations. Idiopathic PAH patients and sex-and-age-matched healthy controls were recruited at Hospital Universitario de la Princesa in Madrid. The relevant clinical characteristics of the patients are summarized in **Table 1**. We have followed the Organic Law 3/2018, December 5, on the Protection of Personal Data, the European Regulation (EU) 2016/679 on data protection as well as the rest of implementing regulations, and the provisions in this regard contemplated in Law 41/2002, of November 14, regulating the autonomy of the patient and rights and obligations in terms of information and clinical documentation, and 14/2007 of Biomedical Research. A code was assigned to the blood obtained for the study, so that it cannot be related to the patient. In addition, all the patients signed the consent to participate in the study.

**Table 1:** Characterization of the cohort of patients with PAH.

Age (years)	Gender	mPAP (mmHg)	proBNP (pg/ml)	SMWT (m)	Treatment with pulmonary vasodilator agents	Comorbidities
75	F	30	222	240	Bosentan	Hypercholesterolemia, glaucoma
59	F	52	3,424	180	Sildenafil, Macitentan	No
62	M	52	2,542	280	Sildenafil, Bosentan	Scleroderma, chronic hepatopathy, dyslipidemia, varicose veins, prostatism
77	F	45	3,044	–	No	Arterial hypertension, diabetes, dyslipidemia, ischemic cardiopathy, auricular fibrillation, Gilbert’s syndrome
74	F	Severe (diagnosed by echocardiography)	4,064	–	No	Arterial hypertension, mitral stenosis, diabetes, dyslipidemia auricular fibrillation, pacemaker, chronic renal disease, multiple myeloma

mPAP, mean pulmonary artery pressure; proBNP, pro-brain natriuretic peptide; SMWT, six-minute walk test.

Peripheral blood mononuclear cells (PBMCs) were obtained from human peripheral blood samples by Ficoll gradient and resuspended in RPMI1640 10% FBS medium. Monocytes were depleted from PBMCs by 1-h adhesion to 100 mm plastic dishes. After this process, B cells were negatively isolated by magnetic separation (BioLegend).

B cells were cultured in p96-dishes (200,000 cells/well) for 72 h in RPMI1640 10% FBS and activated with 5 μg/ml anti-IgM (BioLegend), 5 μg/ml anti-CD40 (BioLegend) and, if required, with 10 μg/ml anti-PSGL-1 (KPL1; BioLegend).

### Flow Cytometry

#### Mouse Cells

Different antibody panels were designed for the identification of different peripheral blood, spleen, peritoneal cells, and bone marrow immune cell populations (**Tables 2** and **3**).

**Table 2:** Flow Cytometry Antibodies for characterization of murine immune cells.

Target	Fluorochrome	Dilution	Company	Reference
B220	APC	1:100	BD Pharmingen	553092
CD3	PE-Cy7	1:100	eBioscience	25-0031-82
CD8	PerCP	1:25	Miltenyi	130-102-468
CD4	PE	1:50	Immunotools	22150044
CD49b	APC-Vio770	1:25	Miltenyi	130-105-249
CD11c	PE-Cy7	1:100	eBioscience	25-0114-82
CD11b	FITC	1:100	BD Pharmingen	553310
PSGL-1	BV510	1:100	BD Pharmingen	563448
MCH-II	APC	1:25	Miltenyi	130-102-139
B220	APC-Vio770	1:25	Miltenyi	130-102-267
IgD	Biotinylated	1:25	Miltenyi	130-111-494
IgA	PE	1:50	eBioscience	12-5994-81
IgG	APC	1:50	BioLegend	405308
IgM	FITC	1:25	Miltenyi	130-095-906
CD19	VioBlue	1:25	Miltenyi	130-102-451
CD21	PE-Cy7	1:25	Miltenyi	130-102-200
CD23	FITC	1:25	Miltenyi	130-102-524
CD5	APC	1:25	Miltenyi	130-106-204
CD43	PE	1:25	Miltenyi	130-105-379
CD117 (c-KIT)	PE-Vio770	1:25	Miltenyi	130-108-384
CD127 (IL-7R)	PE	1:25	Miltenyi	130-102-984
CD135 (FLT3)	APC	1:25	Miltenyi	130-111-152
Sca-1	FITC	1:25	Miltenyi	130-102-831
Lineage Cocktail: antibodies against Gr-1 (Ly6G/C), Ter-119, CD5, CD11b, CD45R and Anti-7-4	Biotinylated	1:11	Miltenyi	130-092-613
PSGL-1	PE	1:100	BD Pharmingen	555306
CD138	PE-Vio770	1:25	Miltenyi	130-102-318

**Table 3:** Flow cytometry patterns used to define murine immune populations.

Population	Markers/Criteria	Location
T cells	CD3^+^B220*^−^*	Peripheral blood
B cells	B220^+^CD3*^−^*	
CD4+ T cells	CD3^+^B220*^−^*CD4^+^CD8*^−^*	
CD8+ T cells	CD3^+^B220*^−^*CD4*^−^*CD8^+^	
NK cells	CD3*^−^*B220*^−^*CD49b^+^	
Granulocytes	Low size, high complexity	
Monocytes	High size, low complexity and CD11b^+^	
Dendritic cells	MHC-II^+^CD11c^+^	
LSK	Lin*^−^*Sca-1^+^cKIT^+^	Bone marrow
CLP	Lin*^−^*FLT3^+^IL-7R^+^	
Pre-pro B cell	B220^+^CD19*^−^*cKIT^+^FLT3^+^	
Pro B cell	B220^+^CD19^+^IgD*^−^*IgM*^−^*cKIT^+^FLT3*^−^*	
Pre B cell	B220^+^CD19^+^IgD*^−^*IgM*^−^*cKIT*^−^*FLT3*^−^*	
Immature B cell	B220^+^CD19^+^IgD^+^IgM^+^	
Mature B cell	B220^+^CD19^+^IgD*^−^*IgM^+^	
Plasma cell	CD138^high^	
IgA+ B cells	B220^+^IgA^+^	Spleen
IgM+ B cells	B220^+^IgA*^−^*IgM^+^IgD*^−^*	
IgM+IgD+ B cells	B220^+^IgA*^−^*IgM^+^IgD^+^	
IgD+ B cells	B220^+^IgA*^−^*IgM*^−^*IgD^+^	
IgG+ B cells	B220^+^IgA*^−^*IgM*^−^*IgD*^−^*IgG^+^	
B1a cells	CD19^+^IgM^+^CD43^+^CD23^−^CD5^+^	
B1b cells	CD19^+^IgM^+^CD43^+^CD23^−^CD5*^−^*	
Regulatory B cells	B220^+^CD43*^−^*CD5^+^CD21^+^	
Marginal zone B cells	B220^+^CD43*^−^*CD5*^−^*CD21^+^CD23*^−^* ^/dim^	
Follicular B cells	B220^+^CD43*^−^*CD5*^−^*CD21^dim^CD23^+^	
Plasma cells	CD138^high^	
B1a cells	CD19^+^IgM^+^CD23^−^CD5^+^	Peritoneal fluid
B1b cells	CD19^+^IgM^+^CD23^−^CD5*^−^*	
B2 cells	CD19^+^IgM^+^CD23^+^CD5*^−^*	

Briefly, after incubation with 1:200 Fc Block (BD Pharmingen), cells were stained with the cocktail of antibodies targeting surface molecules for 15 min at 4°C. Then, cells were washed and incubated with PerCP-conjugated streptavidin (if necessary) (BD Pharmingen). Subsequently, cells were fixed and permeabilized with 2 ml of FACS Lysing Solution (BD Pharmingen) for 15 min. This solution was also required for erythrocyte lysis. After washing, 5 μl of counting beads were added to each tube for the assessment of the absolute number of cells present in the sample. Finally, samples were acquired and analyzed with a FACSCanto II and FACS Diva software.

#### Human B Cells

Similarly, human B cells were washed and blocked with 1 mg/ml human gammaglobulin. Then, cells were incubated with the cocktail of antibodies targeting surface molecules for 10 min in the dark at 4°C. Cells were washed, permeabilized with FACS Lysis (BD Pharmingen), and incubated for 30 min with the anti-IL-10 antibody. **Tables 4** and **5** show the list of human antibodies and the strategy used for B cell subset identification.

**Table 4:** Flow cytometry antibodies for characterization of human immune cells.

Target	Fluorochrome	Dilution	Company	Reference
CD19	V500	1:50	BD Horizon	561121
CD27	APC-H7	1:50	BD Pharmingen	560222
IgA	APC	1:50	Miltenyi	130-093-113
IgG	FITC	1:50	BD Pharmingen	555786
PSGL-1	PE	1:50	BD Pharmingen	556055
IgD	FITC	1:50	BD Pharmingen	555778
IgM	PerCP-Cy5.5	1:50	BD Pharmingen	561285
CD3	V500	1:50	BD Horizon	561416
CD14	V500	1:50	BD Horizon	561391
CD123	APC	1:50	BioLegend	306012
CD16	APC	1:50	BioLegend	302012
CD38	APC	1:50	BioLegend	303510

**Table 5:** Flow cytometry patterns used to characterize PSGL-1 expression on different CD27^+^ and CD27*^−^* B cells subpopulations.

Population	Markers/Criteria	Culture Day
CD27^+^IgA^+^ B cells	CD19^+^CD27^+^IgA^+^IgG*^−^*	Day 0
CD27^+^IgG^+^ B cells	CD19^+^CD27^+^IgG^+^IgA*^−^*	
CD27^+^IgM^+^ B cells	CD19^+^CD27^+^IgM^+^IgD*^−^*	Day 0
CD27^+^IgD^+^ B cells	CD19^+^CD27^+^IgD^+^IgM*^−^*	
CD27^+^IgD^+^IgM^+^ B cells	CD19^+^CD27^+^IgD^+^IgM^+^	
CD27*^−^*IgA^+^ B cells	CD19^+^CD27*^−^*IgA^+^IgG*^−^*	Day 0
CD27*^−^*IgG^+^ B cells	CD19^+^CD27*^−^*IgG^+^IgA*^−^*	
CD27*^−^*IgM^+^ B cells	CD19^+^CD27*^−^*IgM^+^IgD*^−^*	Day 0
CD27*^−^*IgD^+^ B cells	CD19^+^CD27*^−^*IgD^+^IgM*^−^*	
CD27*^−^*IgD^+^IgM^+^ B cells	CD19^+^CD27*^−^*IgD^+^IgM^+^	
B cells	CD3*^−^*CD14*^−^*CD123*^−^*CD16*^−^*CD27*^−^* ^/+^	Day 3
CD27^+^IgG^+^ B cells	CD3*^−^*CD14*^−^*CD123*^−^*CD16*^−^*CD27^+^IgG^+^	
CD27^+^IgD^+^ B cells	CD3*^−^*CD14*^−^*CD123*^−^*CD16*^−^*CD27^+^IgD^+^	
CD27*^−^*IgG^+^ B cells	CD3*^−^*CD14*^−^*CD123*^−^*CD16*^−^*CD27*^−^*IgG^+^	
CD27*^−^*IgD^+^ B cells	CD3*^−^*CD14*^−^*CD123*^−^*CD16*^−^*CD27*^−^*IgD^+^	

For viability assays, 7-AAD (7- Amino Actinomycin D), was used. Briefly, after the staining process, cells were incubated with 1:500 7-AAD for 5 min in the dark.

At day 0, B cells were identified as CD19^+^ cells. At day 3, CD19 was notably down-regulated; therefore, a negative exclusion strategy was used: CD3 was used to exclude T cells, CD14 for monocytes, CD123 for basophils and dendritic cells (DC), and CD16 for neutrophils and NK cells.

For membrane PSGL-1 evaluation, incubation with surface markers including anti-PSGL-1 antibody was performed as described above. For total PSGL-1 expression measurement, after the staining with surface markers, cells were fixed and permeabilized with FACS Lysis (BD Pharmingen), and incubated with the anti-PSGL-1 antibody for 30 min at 4°C in the dark. For peripheral blood plasma cell identification, cells were first gated by intermediate forward and side scatter position, then selected CD19^+^CD3^−^ cells and plasma cells were identified as CD38^++^ criteria in the CD19^+^ gated cells (**Supplementary Figure 1**).

### Immunophenotyping of PSGL-1 KO Mice

For *PSGL-1^−/−^* mice immunophenotyping, the Ig Isotyping Mouse Instant ELISA Kit (Invitrogen) for qualitative detection of mouse Ig isotypes was used. WT and *PSGL-1^−/−^* sera were diluted 1:3,000 with 0.9% NaCl before added to the kit. Manufacturer´s instructions were followed for Ig detection. Absorbance at 450 nm was measured in a Glomax multidetection system fluorimeter (Promega).

### Statistical Analysis

Two-tailed Student’s t-test or Mann-Whitney U test were used for comparison of groups depending on whether data had a normal or non-normal distribution. Three-group comparison was performed using one-way analysis of variance (ANOVA) with the Bonferroni *post hoc* test for parametric variables and Kruskal-Wallis test for non-parametric variables. All statistical analyses were performed using GraphPad Prism 6 (GraphPad Software).

## Results

### Characterization of Blood Cell Populations in *PSGL-1^−/−^* Mice and Analysis of PSGL-1 Expression in Circulating Cells

**Figure 1:**
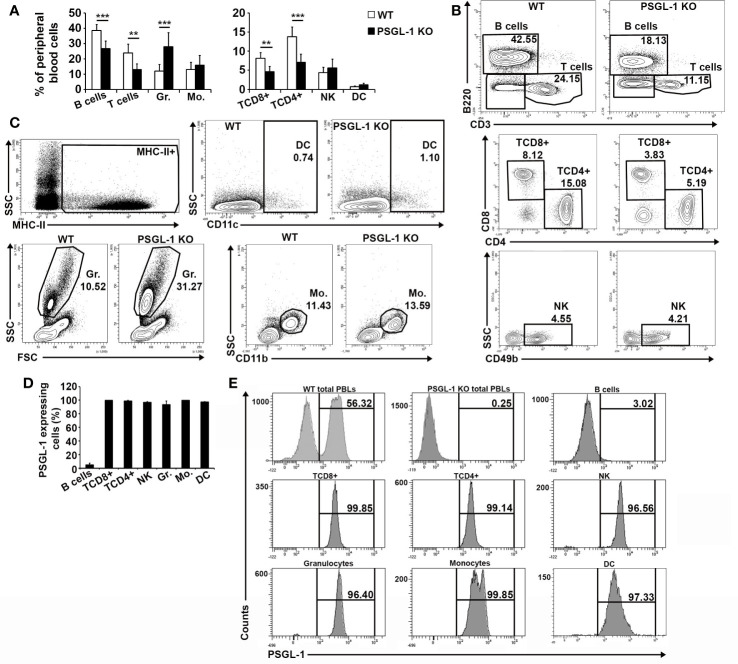
Characterization of blood cell populations in *PSGL-1^−/−^* mice and analysis of PSGL-1 expression in circulating cells. **(A)** Relative frequencies of the circulating immune cell populations in WT and *PSGL-1^−/−^* mice. **(B)** Representative density plots for WT and *PSGL-1^−/−^* mice showing gating strategy for B (B220^+^) and T cells (CD3^+^) (upper panels); CD4^+^ T cells (TCD4+) and CD8^+^ T cells (TCD8+) gated in the CD3^+^ population (middle panels); natural killer cells (NK) (CD49b^+^) gated in the CD3*^−^*B220*^−^* double negative population (lower panels). **(C)** Representative dot/density plots for WT and *PSGL-1^−/−^* mice showing gating strategy for: granulocytes (Gr.) identified by size and complexity; dendritic cells (DC) (CD11c^+^MHC-II^+^); and monocytes (Mo.) (CD11b^+^) of WT and *PSGL-1^−/−^* mice. **(D)** Percentage of PSGL-1 expressing cells among the different circulating immune populations of WT mice. **(E)** Representative histograms showing PSGL-1 expression in the above-mentioned circulating immune cell populations. Bars represent the mean+standard deviation. **p < 0.01, ***p < 0.001 analyzed by Student’s t test. In all cases, n = 6 mice per group.

Previous studies in our laboratory described that peripheral blood immune populations in *PSGL-1^−/−^*mice presented increased percentage of circulating myeloid cells and lower frequencies of circulating lymphoid cells compared to WT mice (29). In the present work, the lymphoid compartment was deeply analyzed in WT and *PSGL-1^−/−^* mice. No statistically significant differences were found in the NK population between WT and *PSGL-1^−/−^*mice, whereas both B and T cell subsets were clearly reduced in *PSGL-1^−/−^* mice. Within the T cell subset, both CD4^+^ and CD8^+^ T cell frequencies were reduced in the blood of *PSGL-1^−/−^* mice (**Figures 1A–C**).

In addition, the expression of PSGL-1 was also analyzed in the different populations of circulating immune cells of WT mice. In granulocytes, T cells, monocytes, NK cells, and DCs, the percentage of PSGL-1^+^ cells was close to 100% (**Figures 1D, E**). In contrast, the mean percentage of B lymphocytes expressing PSGL-1 (4.7 ± 0.8) was drastically lower compared to the rest of immune cell subsets (**Figures 1D, E**).

### B Cell Generation Is Impaired in the Bone Marrow of *PSGL-1^−/−^*Mice

**Figure 2:**
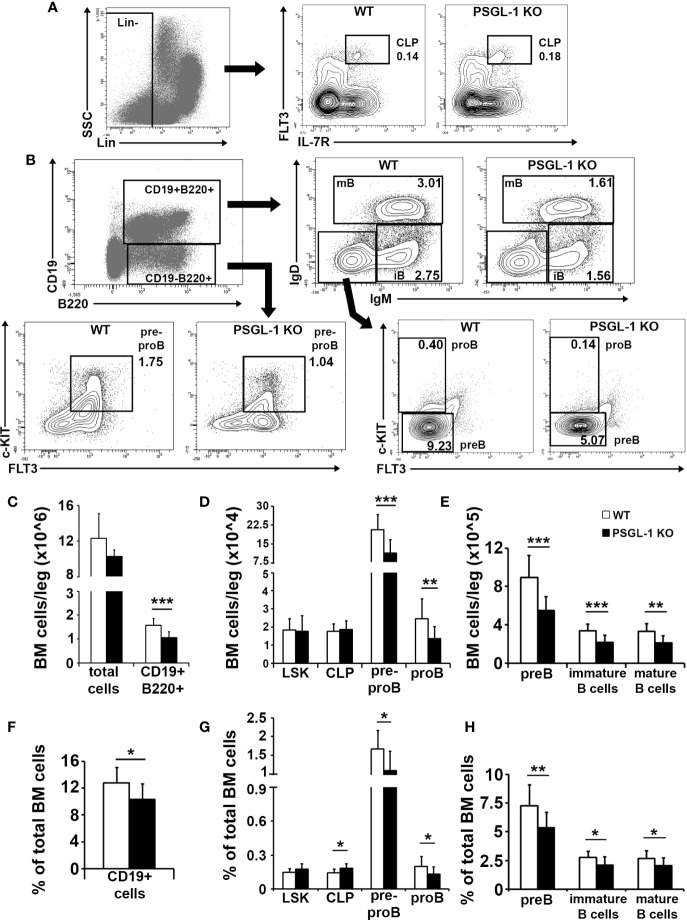
B cell generation is impaired in the bone marrow of *PSGL-1^−/−^* mice. **(A)** Gating strategy for bone marrow lineage negative (Lin*^−^*) gating (left panel) and representative dot/density plots of common lymphoid progenitors (CLP) of WT and *PSGL-1^−/−^* mice (middle and right panels). **(B)** Gating strategy and representative dot/density plots showing bone marrow pre-pro B, pro-B, pre-B, immature (iB), and mature (mB) B cells of WT and *PSGL-1^−/−^* mice. **(C–E)** Total number of cells of the following populations in the bone marrow of WT and *PSGL-1^−/−^* mice: CD19^+^B220^+^ B cell lineage-committed cells **(C)**; Lin*^−^*Sca-1^+^c-KIT^+^ (LSK), CLP, pre-pro B and pro-B **(D)**; pre-B, immature B cells and mature B cells **(E)**. **(F–H)** Relative frequencies of the following populations in the bone marrow of WT and *PSGL-1^−/−^* mice: CD19^+^B220^+^ B cell lineage-committed cells **(F)**; LSK, CLP, pre-pro B and pro-B **(G)**; pre-B, immature B cells, and mature B cells **(H)**. Bars represent the mean+standard deviation. *p < 0.05, **p < 0.01, ***p < 0.001 analyzed by Student’s t test. In all cases, n = 12 mice per group.

Given the reduction of circulating B cells in *PSGL-1^−/−^* mice, a possible alteration of B cell generation in the bone marrow was analyzed (**Figures 2A, B**). Total number of cells in the bone marrow was similar in WT and *PSGL-1^−/−^*mice, although the absolute number and percentage of the CD19^+^B220^+^ B cell-committed compartment was reduced in *PSGL-1^−/−^* mice (**Figures 2C, F**). Lin*^−^*Sca-1^+^c-KIT^+^ compartment (LSK), which include HSC and the most immature multipotent precursors, and Lin*^−^*FLT3^+^IL-7R^+^ CLP were studied among lineage negative bone marrow progenitors (Lin*^−^*). Although similar numbers of LSK and CLP were found in WT and *PSGL-1^−/−^* mice, the percentage of CLP was increased in the bone marrow of *PSGL-1^−/−^*mice (**Figures 2A, D, G**). The analysis of the different B cell-lineage committed subpopulations showed a significant reduction in the number and percentage of pre-pro B (CD19*^−^*B220^+^c-KIT^+^FLT3^+^), pro-B (CD19^+^B220^+^IgD*^−^*IgM*^−^*FLT3*^−^*c-KIT^dim^), pre-B (CD19^+^B220^+^IgD*^−^*IgM*^−^*FLT3*^−^*c-KIT*^−^*), immature B cell (CD19^+^B220^+^IgD*^−^*IgM^+^), and mature B cell (CD19^+^B220^+^IgD^+^IgM^+^) subsets (**Figures 2B, D, E, G, H**).

### PSGL-1 Expression Is Gradually Down-Regulated Throughout B Cell Development

**Figure 3:**
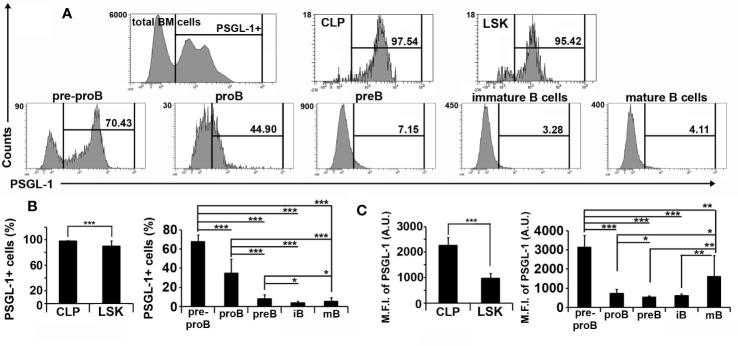
PSGL-1 expression is gradually down-regulated throughout B cell development. **(A)** Representative histograms showing PSGL-1 expression in total bone marrow cells, Lin*^−^*Sca-1^+^c-KIT^+^ (LSK), common lymphoid progenitors (CLP), pre-pro B cells, pro-B cells, pre-B cells, immature (iB) B cells, and mature (mB) B cells of WT mice. **(B, C)** Percentage of PSGL-1 expressing cells **(B)** and PSGL-1 mean fluorescence intensity (MFI) **(C)** among the different bone marrow precursors and B cell lineage-committed subsets of WT mice. Bars represent the mean+standard deviation. *p < 0.05, **p < 0.01, ***p < 0.001 by Kruskal-Wallis test. In all cases, n = 12 mice per group.

PSGL-1 expression in bone marrow cells of WT mice was also characterized. Almost the whole LSK and CLP populations expressed PSGL-1, although the percentage of cells expressing PSGL-1 and the expression level of PSGL-1 in LSK were lower compared to CLP (**Figures 3A–C**). Regarding the B cell committed lineage, pre-pro B cells showed the highest percentage of PSGL-1-expressing cells (67.49 ± 7.01) and highest PSGL-1 expression level [mean fluorescence intensity (MFI), 3,127 ± 631; **Figures 3A–C**]. The percentage of PSGL-1^+^ cells decreased gradually as B-cell development progressed: pro-B (34.66 ± 14.79), pre-B (7.80 ± 4.74), immature B cells (3.78 ± 1.82), and mature B cells (5.29 ± 4.02) (**Figures 3A–C**). The analysis of PSGL-1 expression in developing bone marrow B cells showed that PSGL-1 MFI was drastically reduced in the transition from pre-pro B to pro-B stages, and was partially recovered in the mature B cell stage (**Figures 3A–C**).

### PSGL-1 Could Modulate Immunoglobulin Expression on B Cells in the Spleen

**Figure 4:**
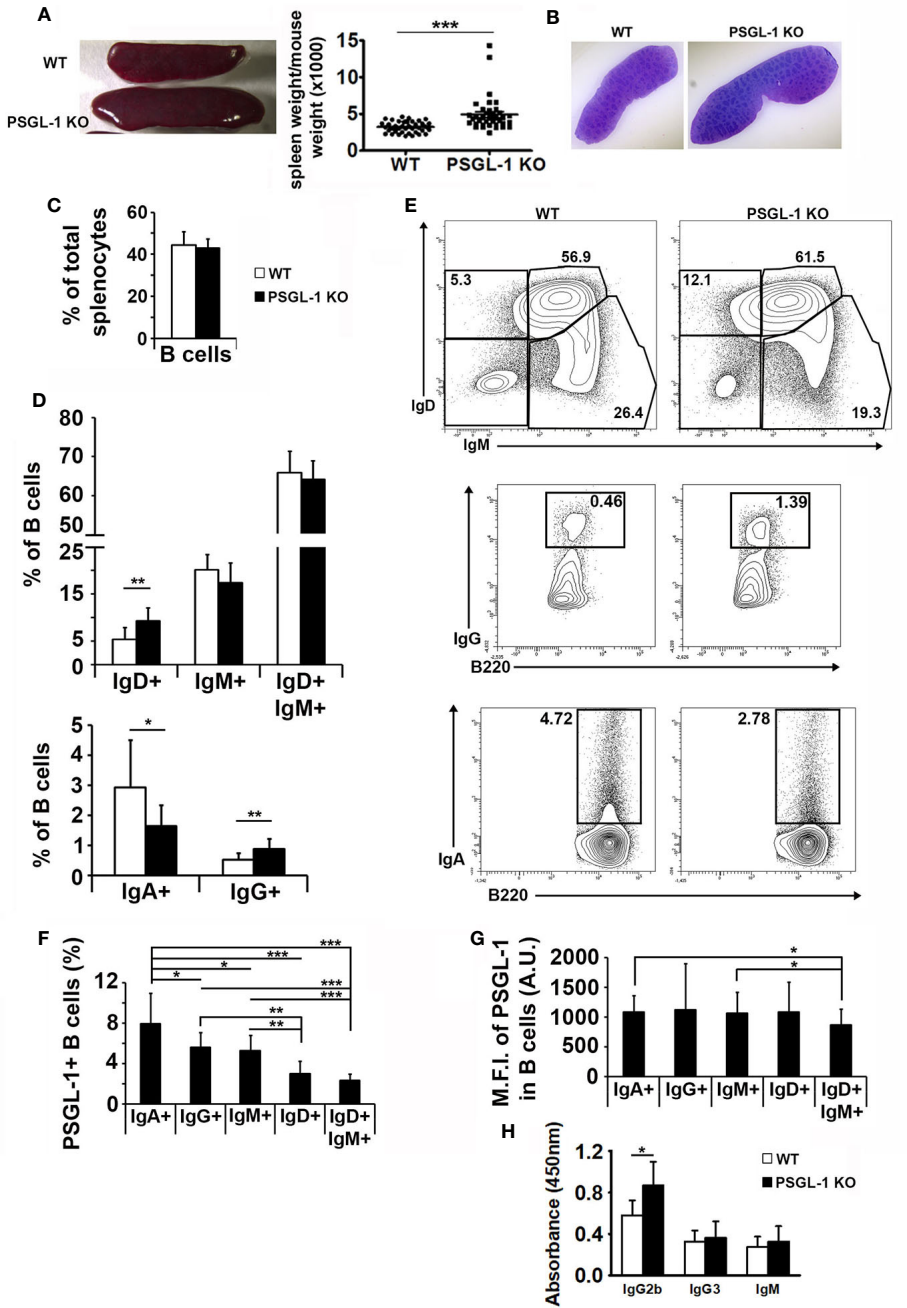
PSGL-1 could modulate immunoglobulin expression on B cells. **(A)** Left panel: image showing the spleens of 3-month-old WT (above) and *PSGL-1^−/−^* mice (below). Right panel: spleen weight/body weight ratio in WT (n = 40) and *PSGL-1^−/−^*mice (n = 37). Bars represent the mean+SEM. **(B)** Representative hematoxylin and eosin sections of the spleen of WT and *PSGL-1^−/−^*mice. **(C)** Percentage of splenic B cells in WT and *PSGL-1^−/−^*mice. **(D)** Percentage of splenic immunoglobulin expressing B cells in WT and *PSGL-1^−/−^*mice. **(E)** Representative density plots showing splenic immunoglobulin expressing B cells in WT and *PSGL-1^−/−^* mice: IgM^+^IgD^+^ naive B cells, IgM^+^ and IgD^+^ B cells (upper panels); IgA^+^ B cells (lower panels); IgG^+^ B cells (middle panels). **(F, G)** Percentage of PSGL-1 expressing cells **(F)** and PSGL-1 mean fluorescence intensity (MFI) **(G)** in different WT splenic B cell subsets expressing IgA, IgG, IgM, IgD, or IgM and IgD. In all cases, n = 10–12 mice/group. Bars represent the mean+standard deviation. *p < 0.05, **p < 0.01, ***p < 0.001 by Kruskal-Wallis test. **(H)** IgG2b, IgG3 and IgM serum levels in WT and *PSGL-1^−/−^* mice (n = 5 mice/group). Bars represent the mean+standard deviation. *p < 0.05 by Student’s T test.

Since we found that PSGL-1 deficiency reduces B cell generation in the bone marrow, spleen structure and splenic B lymphocyte populations were analyzed. Spleens were bigger in *PSGL-1^−/−^*mice than in WT mice (**Figures 4A, B**). However, the histological analysis of the spleen revealed similar tissue structure in WT and *PSGL-1^−/−^*mice (**Figure 4B**) and the percentage of B220^+^ B cells in total spleen cells was similar in the two groups of mice (**Figure 4C**).

Importantly, the analysis of the membrane immunoglobulins expressed by splenic B cells in WT and *PSGL-1^−/−^* mice revealed that *PSGL-1^−/−^*mice presented higher percentages of IgD^+^ and IgG^+^ B cells and reduced frequency of IgA^+^ B cells in the spleen, but similar percentage of IgM^+^ B cells and naïve B cells (IgD^+^ IgM^+^) (**Figures 4D, E**).

Moreover, the results obtained with WT mice showed that the percentage of PSGL-1^+^ cells was different in B cell subsets expressing different types of surface immunoglobulin. IgA^+^ B cells presented the highest percentage of PSGL-1^+^ cells (7.94 ± 1.98), followed by IgG^+^ (5.60 ± 0.58), IgM^+^ (5.29 ± 0.86), IgD^+^ (3.01 ± 0.60), and IgD^+^IgM^+^ subsets (2.35 ± 0.54). The differences were statistically significant in all the cases except for IgD^+^ and double positive B cells (**Figure 4F**). However, PSGL-1 expression levels were similar in almost all the B cell subpopulations regardless of the type of surface immunoglobulin they expressed (**Figure 4G**). Statistically significant differences were found in the MFI of PSGL-1 between IgA^+^ or IgM^+^ B cells and IgD^+^IgM^+^ B cells. In agreement with the higher frequency of IgG^+^ B cells present in the spleen of *PSGL-1^−/−^* mice, significantly higher levels of IgG2b were found in blood serum of *PSGL-1^−/−^* mice. The levels of IgG3 and IgM were similar between WT and *PSGL-1^−/−^* mice (**Figure 4H**).

The frequencies of plasma cells in total spleen and bone marrow cells showed no difference between WT and *PSGL-1^−/−^* mice (**Supplementary Figures 2A, B**). In contrast with B cells, more than 80% of WT spleen and bone marrow plasma cells expressed PSGL-1 (**Supplementary Figures 2C, E**). Interestingly, plasma cells in the bone marrow expressed higher PSGL-1 levels than plasma cells in the spleen (**Supplementary Figures 2D, E**).

### Analysis of B1 and B2 Cell Populations in the Spleen and Peritoneal Cavity of WT and *PSGL-1^−^* ^/^ *^−^* Mice

**Figure 5:**
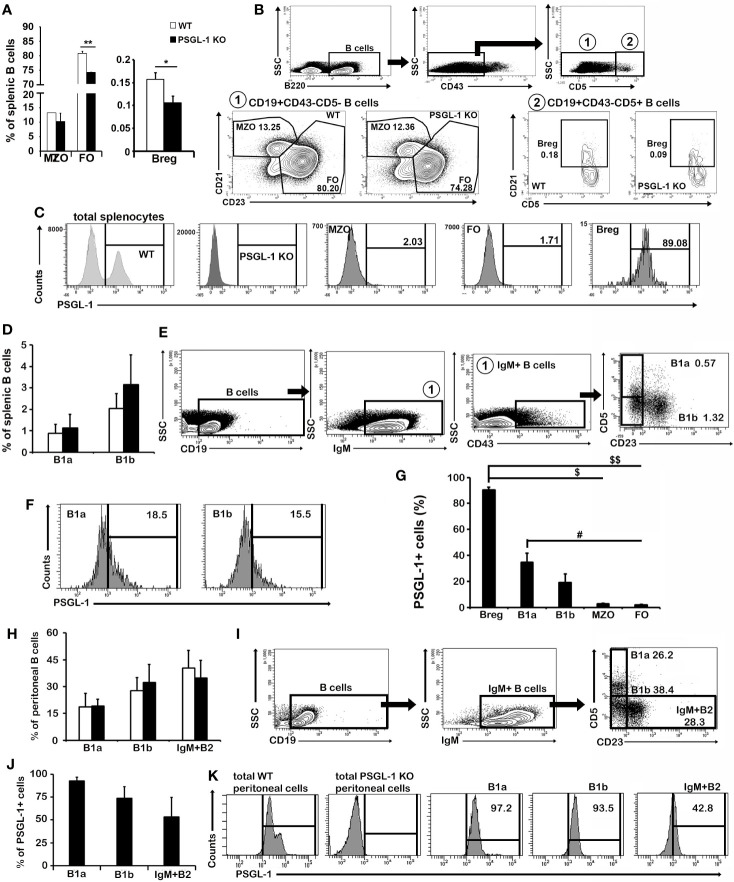
PSGL-1 is differentially expressed in splenic and peritoneal B1 and B2 cells. **(A)** Relative frequencies of the B2 different subsets of marginal zone (MZO), follicular (FO), and regulatory B cells (Breg) in the spleen of WT and *PSGL-1^−/−^*mice. **(B)** Gating strategy and representative density plots showing Breg, FO, and MZO B cells of WT and *PSGL-1^−/−^*mice. **(C)** Representative histograms showing the expression of PSGL-1 in total spleen cells and in the different subsets of B2 cell subpopulations studied in WT and *PSGL-1^−/−^* mice. **(D)** Relative frequencies of B1a and B1b cell subsets in the spleen of WT and *PSGL-1^−/−^* mice. **(E)** Gating strategy and representative density and dot plots showing splenic B1a and B1b cell subsets. **(F)** Representative histograms showing the expression of PSGL-1 in WT B1a and B1b cells. **(G)** Percentage of PSGL-1 expressing cells of different subsets of B1 and B2 cell subpopulations. **(H)** Relative frequency of B1 and IgM^+^B2 cells in the peritoneal cavity of WT and *PSGL-1^−/−^* mice. **(I)** Gating strategy and representative density and dot plots showing peritoneal B1a and B1b cell subsets. **(J, K)** Percentage of PSGL-1^+^ cells **(J)** and PSGL-1 expression representative histograms **(K)** of peritoneal B1a, B1b, and IgM^+^B2 cells. Bars represent the mean+standard deviation. **(A)** Breg *p < 0.05, FO **p < 0.01; **(G)** Breg: ^$^p < 0.05, ^$$^p < 0.01, B1a: ^#^p < 0.05; by Kruskal-Wallis test. In all cases, n = 4–6 mice per group.

To further characterize the alterations in the splenic B cell population, the frequencies of the B2 [follicular (FO), marginal zone (MZO), and Breg] and B1 (B1a and B1b) cells were evaluated (**Figures 5A, B, D, E**). Among B2 cells, a reduced percentage of FO B cells (B220^+^CD43*^−^*CD5*^−^*CD21*^−^*CD23^+^) was found in *PSGL-1^−/−^*mice, whereas the percentage of MZO B cells (B220^+^CD43*^−^*CD5*^−^*CD21^+^CD23^+^) was similar between WT and *PSGL-1^−/−^*mice (**Figures 5A–C**). Interestingly, the Breg population (B220^+^CD43*^−^*CD5^+^CD21^+^) was reduced in the spleens of *PSGL-1^−/−^*mice (**Figures 5C, G**). No statistically significant differences in the proportions of either B1a (CD19^+^IgM^+^CD43^+^CD23*^−^*CD5^+^) or B1b cells (CD19^+^IgM^+^CD43^+^CD23^−^CD5^−^) were found between WT and *PSGL-1^−/−^*mice, although there was a tendency to higher presence of both populations in *PSGL-1^−/−^*mice (**Figures 5D, E**).

Of note, Breg showed the highest percentage of PSGL-1^+^ cells (90%). The percentages of PSGL-1^+^ cells among B1a and B1b cells reached 34.67 ± 6.92% and 19.10 ± 6.73%, respectively (**Figures 5C, F, G**). However, the percentage of PSGL-1^+^ cells was significantly lower in B2 cells than in Breg (**Figure 5G**). B1a cells presented a higher percentage of PSGL-1^+^ cells compared with FO B cells (**Figure 5G**).

PSGL-1 expression was also studied in the resident peritoneal fluid B1 cell population. As around 30% of B1 cells lose the expression of CD43 peritoneal (32), B1a cells were gated as CD19^+^IgM^+^CD23^−^CD5^+^ and B1b cells were gated as CD19^+^IgM^+^CD23^−^CD5^−^ (**Figure 5I**). On the other hand, IgM^+^B2 cells were gated as CD19^+^IgM^+^CD23^+^CD5^-^ (**Figure 5I**). Strikingly, although we did not find differences in the frequencies of these populations between WT and *PSGL-1^−/−^* mice (**Figure 5H**), the percentages of PSGL-1^+^ B1a, B1b and B2 cells were higher in the peritoneal fluid populations than in their splenic counterparts (**Figures 5J, K**): 92.45 ± 4.06% of B1a cells and 73.80 ± 12.68% of B1b cells expressed PSGL-1, whereas B2 cells showed 53.37 ± 21.25% of PSGL-1 expressing cells (**Figures 5J, K**).

### PSGL-1 Signaling Inhibits *In Vitro* Activation of Human Peripheral B Cells

**Figure 6:**
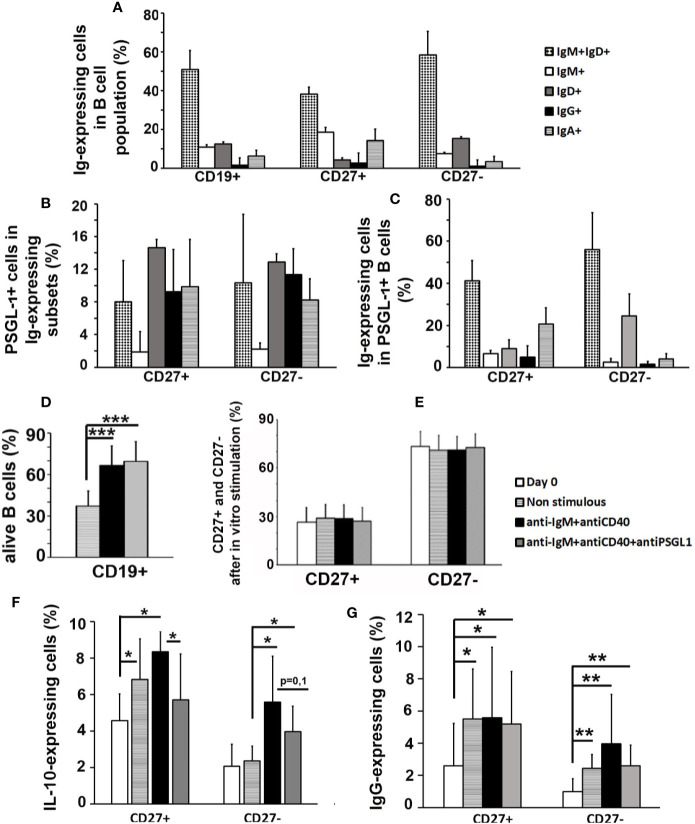
PSGL-1 signaling inhibits *in vitro* activation of human peripheral B cells. **(A)** Relative frequencies of the different immunoglobulin (Ig)-expressing cell subsets in human total B cells (CD19^+^) and in the CD27^+^ and CD27*^−^* B cell subpopulations. **(B)** Percentage of PSGL-1^+^ cells in the different Ig- expressing subsets of CD27^+^ and CD27*^−^* B cells. **(C)** Relative frequencies of the different Ig- expressing cell subsets in the PSGL-1^+^ B cell subpopulation. **(D)** Percentage of surviving B cells after 72 h in culture: non-stimulated, activated with anti-IgM+anti-CD40 antibodies or activated with anti-IgM+anti-CD40+anti-PSGL-1 antibodies. **(E)** Relative frequency of CD27^+^ and CD27*^−^* B cells in the total B cell population after 72 h in culture: non-stimulated, activated with anti-IgM+anti-CD40 antibodies or activated with anti-IgM+anti-CD40+anti-PSGL-1 antibodies. **(F, G)** Percentage of IL-10^+^ **(F)** and IgG^+^ **(G)** cells in CD27^+^ and CD27*^−^* B cells after 72 h in culture: non-stimulated, activated with anti-IgM+anti-CD40 antibodies or activated with anti-IgM+anti-CD40+anti-PSGL-1 antibodies. Bars represent the mean+standard deviation. *p < 0.05, **p < 0.01 by one-way ANOVA test. In all cases, n = 10.

It was recently described that less than 10% of human circulating B cells expressed PSGL-1 in human healthy donors (31). In this work, the proportion of human circulating B cells expressing different types of Igs was analyzed in healthy donors and, as expected, most B cells were naïve (IgD^+^IgM^+^) and only a small percentage presented isotype switching (IgG^+^ or IgA^+^) (**Figure 6A**). Interestingly, the proportion of PSGL-1-expressing cells was similar in all subtypes, except for IgM^+^ B cells which contained a very small percentage of PSGL-1-expressing cells (**Figure 6B**). Of note, compared to the total B cell population, the PSGL-1-expressing B cell subpopulation showed increased percentage of IgD^+^, IgG^+^, and IgA^+^ B cells and reduced IgM^+^ B cells (**Figure 6C**).

To analyze whether PSGL-1 could modulate the activation of B cells, peripheral blood B lymphocytes isolated from healthy donors were incubated *in vitro* with anti-IgM and anti-CD40 antibodies. The presence of an PSGL-1-agonist antibody during a 3-day culture of B cells with anti-IgM+anti-CD40 activation showed that PSGL-1 signaling in B cells did not affect B cell survival (**Figure 6D**) or the balance between CD27^+^ (some mature unswitched and most of switched B cells) and CD27*^−^* B cells (naïve B cells and a minority of switched mature B cells) (**Figure 6E**), but reduced the frequency of IL-10 expressing B cells (**Figure 6F**). In addition, PSGL-1 signaling impaired the increase of IgG^+^ B cells triggered by IgM and CD40 stimulation of the CD27*^−^* subset (**Figure 6G**).

### Membrane PSGL-1 Expression Is Reduced in Circulating Plasma Cells of Patients With Pulmonary Arterial Hypertension

**Figure 7:**
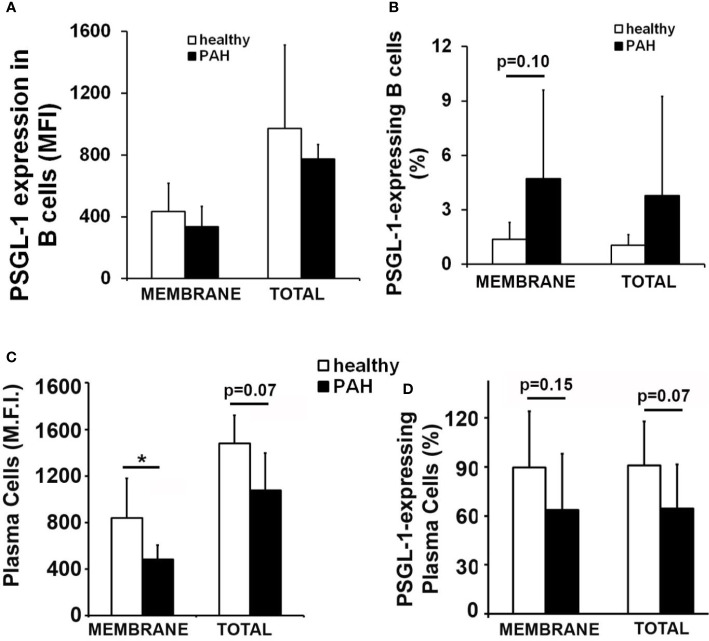
Membrane PSGL-1 expression is reduced in circulating plasma cells of patients with pulmonary arterial hypertension. **(A, B)** Membrane and total expression levels of PSGL-1 **(A)** and percentage of PSGL-1 expressing cells **(B)** in circulating B cells of healthy donors and patients with pulmonary arterial hypertension (PAH). **(C, D)** Membrane and total expression levels of PSGL-1 **(C)** and percentage of PSGL-1 expressing cells **(D)** in circulating plasma cells of healthy donors and patients with PAH. *p < 0.05 by Mann Whitney’s U test. In all cases, n = 7 healthy donors and 5 idiopathic PAH patients.

In this work, given the high incidence of pulmonary hypertension among scleroderma patients, which present reduced expression of PSGL-1 in B cells, membrane and total expression of PSGL-1 was analyzed in circulating B cells and plasma cells of patients with idiopathic PAH. Of note, no difference in B cell PSGL-1 expression level was found between PAH patients and controls, whereas a tendency to a higher percentage of PSGL-1 expressing B cells was observed in these patients (**Figures 7A, B**). However, PAH patients showed reduced expression level of membrane PSGL-1 on plasma cells and a tendency to reduced percentage of circulating plasma cells expressing PSGL-1 (**Figures 7C, D**).

## Discussion

In this work, we characterized the expression of PSGL-1 through different developmental stages of B cell generation and maturation, as well as the consequences of PSGL-1 absence in B cell development and activation to assess whether PSGL-1 expression is regulated during B cell generation. Although the number of LSK and CLP did not change in the absence of PSGL-1, decreased numbers of peripheral blood B cells and bone marrow B cell lineage-committed subpopulations were found in *PSGL-1^−/−^* mice, indicating that PSGL-1 is important for B cell development. In agreement with results described in the literature, we found that PSGL-1 was expressed in 95–99% of LSK and CLP (33, 34), with higher expression levels in CLP. Interestingly, the percentage of PSGL-1^+^ cells decreased to 60% when CLP advanced to the pre-pro B cell stage, although the PSGL-1 expression level remained high in the positive population (**Figure 3**). Additionally, this percentage decreased to 30% in pro-B cells, although in this case PSGL-1 expression level was low in the remaining positive cells, suggesting that PSGL-1 is down-regulated in the progenitors when they commit to the B cell lineage. Moreover, PSGL-1 expression level was maintained low in pre-B, immature B cell and mature B cell developmental stages, in which the percentage of PSGL-1 expressing cells was reduced below 5%, indicating that PSGL-1 is negatively regulated during B cell generation in the bone marrow. Regarding *PSGL-1^−/−^*mice, our data show reduced number and percentage of pre-pro B cells and increased percentage of CLP, suggesting a possible contribution to the expression of genes that control commitment to the B cell lineage. Accordingly, in *PSGL-1^−/−^*mice, cells that reached the pre-proB stage progressed normally throughout the developmental process to become mature B cells. Remarkably, PSGL-1^+^ B cells are part of the Breg population in humans and produce IL-10 (31). In this context, the regulatory pathways would be activated in PSGL-1^+^ cells upon interaction with PSGL-1 ligands, such as L-selectin in other developing leukocytes or E- and P-selectin expressed on the surface of bone marrow stromal cells (31, 34–36). Importantly, the high PSGL-1 expression in plasma cells, through its interaction with P- and E-selectin expressed by bone marrow stromal cells, might help to their homing to the bone marrow (35, 37) and also to regulate antibody production. Accordingly, in the absence of PSGL-1, elimination of autoreactive B cells would be impaired thereby leading to the development of a systemic autoimmune syndrome similar to human scleroderma in adult mice (30). In this line, the lack of PSGL-1 expression in B1 cells, that have been characterized as the main IL-10 producing B cell subset (32, 38), may lead to a reduced IL-10 production and contribute to the systemic autoimmune syndrome in *PSGL-1^−/−^* mice.

Splenomegaly is a common feature observed in mouse models of systemic autoimmunity. In the case of *PSGL-1^−/−^* mice, the increase in the spleen size did not result either in a change of B cell frequency in total spleen cells or a change in spleen structure; although the total number of follicles was higher, their size and structure was similar to follicles in WT mice, suggesting that there is no defect in the arrival of mature naïve B cell to the spleen or in antigen-dependent B cell maturation. However, if autoreactive B cells are not adequately eliminated in the bone marrow of *PSGL-1^−/−^* mice, they could be activated by autoantigens in the spleen, promoting GC reactions and increasing the number of GC. In addition, our data suggest that, in WT mice, PSGL-1 is expressed more frequently in IgA^+^, IgG^+^, and IgM^+^IgD*^−^* splenic subpopulations, which are B cell subpopulations that have undergone CSR. This increment in the percentage of PSGL-1^+^ cells in class switched B cells could reflect the transition to plasmablasts and plasma cells, populations expressing high levels of PSGL-1. In *PSGL-1^−/−^*mice, the frequency of splenic IgA^+^ B cells was reduced, while the frequency of IgD^+^ and IgG^+^ B cells was increased, suggesting that PSGL-1 could favor the switch to IgA and reduce the switch to IgG. Accordingly, PSGL-1^−/−^ mice showed increased serum level of IgG2b. This effect would also be in agreement with the inhibitory effect of an anti-PSGL-1 agonist antibody on IgG generation during *in vitro* activation of human B cells. Remarkably, in the peritoneal B1 cell subset the PSGL-1^+^ population is even higher than in the splenic B1 subset. Interestingly, PSGL-1 expression in WT mice was unexpectedly high in B1 cells and Bregs, compared with the remaining B2 cell subsets (**Figure 5**), suggesting that mouse splenic PSGL-1^+^ B cells, as it happens in human circulating B cells (29), could be part of the Breg population. These cells produce IL-10 in homeostatic conditions, thus exerting a regulatory function, while preventing B cells from apoptosis (39), inducing Ig secretion (40) and regulating the expression of Aicda, a critical component of the CSR machinery (41).

In this work, we also studied the expression of PSGL-1 in human peripheral circulating B cells and found that the frequency of PSGL-1^+^ cells was similar in all the Ig-expressing subsets except for IgM^+^ subpopulation, which showed the smallest percentage of PSGL-1^+^ cells. The most represented subsets among PSGL-1 expressing B cells were naïve, IgA^+^ and IgD^+^ cells, while the most represented subsets in the whole B cell population were naïve, IgM^+^ and IgD^+^ cells. As discussed above, *PSGL-1^−/−^*mice have reduced IgA^+^ and increased IgG^+^ B cell subsets in the spleen, thus suggesting that PSGL-1 could contribute to regulate CSR favoring expression of IgA *versus* IgG. More interestingly, we have found an inhibitory effect of an anti-PSGL-1 agonist antibody on the induction of IL-10^+^ B cells, as well as on the appearance of IgG^+^ cells in the CD27*^−^*B cell subset during *in vitro* activation of human circulating B cells with anti-IgM plus anti-CD40 antibodies. These results suggest that PSGL-1 signaling in B cells may control antibody production by down-regulating IL-10 expression during B cell activation (40).

Our previous work showed that B cells of scleroderma patients have reduced PSGL-1 expression (31). Since the prevalence of PAH is higher in scleroderma patients than in the general population our group also analyzed the PSGL-1 expression in B cells and plasma cells from patients with idiopathic pulmonary arterial hypertension. The results showed evidence that PSGL-1 expression is reduced in plasma cells from PAH patients compared with age-matched healthy donors. These data suggest that the low expression of PSGL-1 in B cells may be responsible of the high incidence of PAH in SSc patients. However, this is an observational study and further research must be performed to confirm our results in a larger cohort of patients. This confirmation would open the possibility of using PSGL-1 as a biomarker of idiopathic PAH and SSc-associated PAH, which would be of great interest given the high PAH prevalence in SSc patients.

## Data Availability Statement

The original contributions presented in the study are included in the article/**Supplementary Material**. Further inquiries can be directed to the corresponding author.

## Ethics Statement

The studies involving human participants were reviewed and approved by Comité de Ética de La Investigación con Medicamentos del Hospital Universitario de la Princesa, N° de Registro: 3106. The patients/participants provided their written informed consent to participate in this study. The animal study was reviewed and approved by Dirección General de Medio Ambiente, Consejería de Medio Ambiente y Ordenación del Territorio de la Comunidad de Madrid. PROEX69-14.

## Author Contributions

AU conceived and supervised the study. RG-T and AU designed and interpreted the experiments and analyzed data. RG-T performed most of the experiments and wrote the manuscript; EG-S, JS, AM-C performed experiments. JG-P, EV-R, and SC gave clinical information and advice and selected patients; SC provided reagents. All authors contributed to the article and approved the submitted version.

## Funding

This work was supported by Ayudas MHER (ayuda Fundación Cajasol 2018) and by Spanish Ministry of Health and Instituto de Salud Carlos III (ISCIII) (cofinanced by European Regional Development Fund, Fondos FEDER) (grant numbers, FIS-PI17-01819, FIS-PI12-01578, AC17-00027).

## Conflict of Interest

The authors declare that the research was conducted in the absence of any commercial or financial relationships that could be construed as a potential conflict of interest.

## Supplementary Material

Supplementary Figure 1 Gating strategy for human plasma cells. Representative dot plots showing the gating strategy to identify the plasma cell population in human peripheral blood.

Supplementary Figure 2 Expression of PSGL-1 in murine plasma cells. **(A)** Representative dot plots showing CD138^high^ plasma cells in the bone marrow and spleen of WT and *PSGL-1^−/−^* mice. **(B)** Relative frequency of plasma cells in the bone marrow and spleen of WT and *PSGL-1^−/−^* mice. **(C, D)** Percentage of PSGL-1^+^ **(C)** and PSGL-1 MFI **(D)** measured in plasma cells (CD138^high^) and total B cells (B220^+^CD19^+^) in the bone marrow and spleen of WT mice. **(E)** Representative histograms showing PSGL-1 expression in bone marrow and spleen cells of WT and *PSGL-1^−/−^* mice. n = 10 mice per group.
