# Supplementary figures and images for: Relevance of PSGL-1 Expression in B Cell Development and Activation
Source: Front Immunol. 2020 Nov 12;11:588212. doi: 10.3389/fimmu.2020.588212 (PMC7689347; doi:10.3389/fimmu.2020.588212)

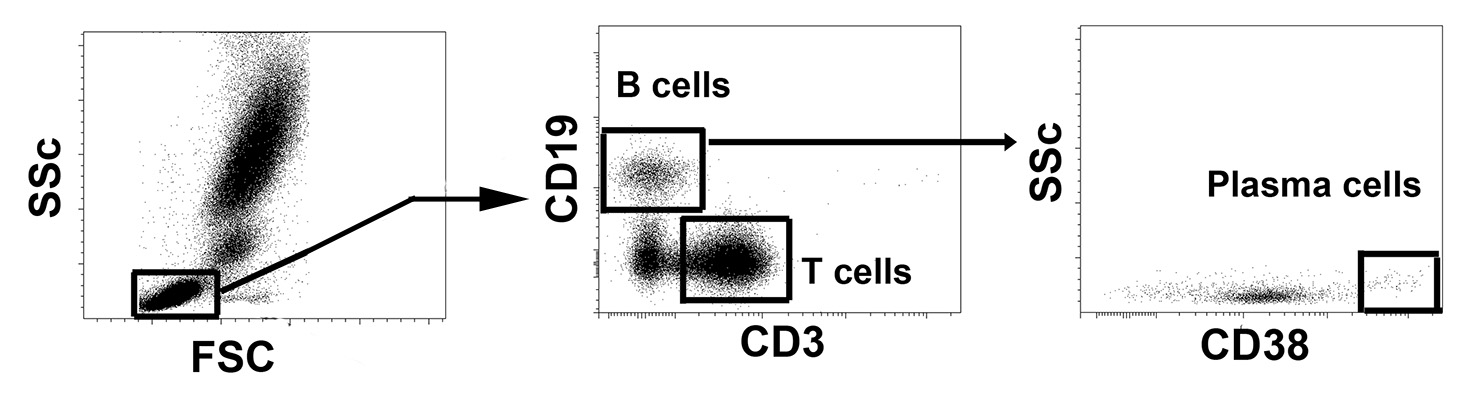

Supplement: Supplementary Figure 1 — Gating strategy for human plasma cells. Representative dot plots showing the gating strategy to identify the plasma cell population in human peripheral blood. [file Image_1.jpeg]

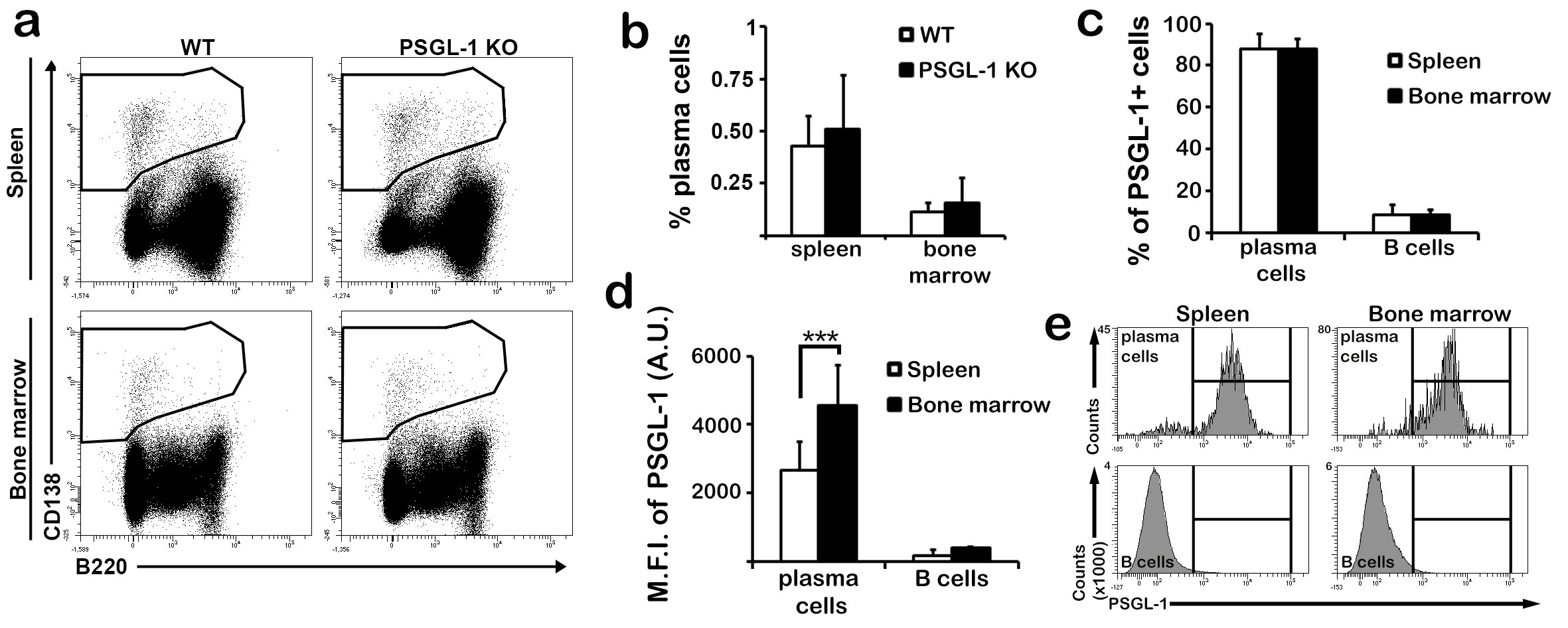

Supplement: Supplementary Figure 2 — Expression of PSGL-1 in murine plasma cells. (A) Representative dot plots showing CD138high plasma cells in the bone marrow and spleen of WT and PSGL-1−/− mice. (B) Relative frequency of plasma cells in the bone marrow and spleen of WT and PSGL-1−/− mice. (C, D) Percentage of PSGL-1+ (C) and PSGL-1 MFI (D) measured in plasma cells (CD138high) and total B cells (B220+CD19+) in the bone marrow and spleen of WT mice. (E) Representative histograms showing PSGL-1 expression in bone marrow and spleen cells of WT and PSGL-1−/− mice. n = 10 mice per group. [file Image_2.jpeg]
